# Revision hip replacement for recurrent Hydatid disease of the pelvis: a case report and review of the literature

**DOI:** 10.1186/1749-799X-5-17

**Published:** 2010-03-11

**Authors:** Venkata SS Neelapala, Coonoor R Chandrasekar, Robert J Grimer

**Affiliations:** 1 The Royal Orthopaedic Hospital, Bristol Road South, Birmingham B31 2AP, UK

## Abstract

A case of a large recurrent hydatid cyst involving the right ilium and right hip treated with excision of the cyst, Total hip replacement and revision of the acetabular component with a Tripolar articulation for cyst recurrence and acetabular component loosening is presented along with a review of the relevant literature. To our knowledge there is no reported case of Total Hip replacement and revision for hydatid disease involving the bony pelvis.

## Introduction

Hydatid disease commonly involves liver and lung. There are many reports on Hydatid disease with the involvement of the musculoskeletal system [1-11]. Involvement of the musculoskeletal system occurs in 1% to 4% of all cases [7]. Hydatid disease is a parasitic infection caused by tapeworm Echinococcus which inhabits in the small intestine of carnivores. The adult worms produce eggs that are released with the faeces and spread in various ways, such as through the wind, water or flies [6]. After ingestion by the host, the embryos migrate through the intestinal wall and are either arrested in the capillary bed of the liver developing into liver cysts, or manage to penetrate into systemic circulation thus ending up in remote organs. Due to their physiologic role as capillary filters and their vast capillary volume, the liver and lung are most often affected. The brain, the muscles or the bones are the more frequently involved distant organs.

In this report, we present a case of a large recurrent hydatid cyst involving the right ilium and right hip treated with excision of the cyst and Total hip replacement which was functional for 80 months and revision with a Tripolar articulation for cyst recurrence and acetabular component loosening followed for 12 months is presented. To our knowledge there is no reported case of Total Hip replacement and revision for hydatid disease involving the bony pelvis.

## Case Report

A 35-year-old female patient who had lived in the United Kingdom all her life was referred with pain in the right side of pelvis in 1997. She was investigated for back and hip problems. All her blood results including inflammatory markers were normal.

Radiographs and bone scan were normal. She was thought to have hip dysplasia and a MRI revealed abnormal signal changes in the right ilium suggestive of neoplasia or infection. She was referred to the Oncology team for further opinion and management in May1998. She was afebrile and there was no history of infections and exposure to Tuberculosis. She did not have any pets and there was no history of contact with farm animals. Biopsy of the pelvic bone was carried out on 11/6/98 and the histology showed necrotic bone with microsequestra surrounded by a foreign body reaction of histiocytes and giant cells with a thin fibrous wall. Special stains for mycobacterium and fungi were negative. Propionibacterium was grown in culture sensitive to penicillin, amoxicillin, erythromycin and ciprofloxacin. She was advised to take of Penicillin-V 500 mg four times a day for six weeks. Nevertheless Propionibacterium bone infection was thought to be the unlikely cause of her hip pain. She was reviewed on 14/12/98 with worsening right hip pain. Examination showed limitation of right hip movements. Radiographs now showed abnormality in the right ilium with narrowing of the joint space. She was advised to have repeat blood tests including a FBC, ESR and Myeloma screen (all the blood tests were normal). MRI scan on 7/1/99 showed altered marrow signal change from inferior part of right sacro-iliac region to acetabulum and lobulated cyst in the soft tissue around the right hip region. Hydatid disease was considered as a diagnosis and Core biopsy of the abnormal region and aspiration of the cysts lateral to the ilium were performed on 28/1/99 and samples were sent for histology, microbiology and hydatid immunotests. Histology suggested cyst with laminar wall, reaction to non-human tissue and inflammation not typical of an abscess. Cultures had grown coagulase negative staphylococci. ELISA test was positive for hydatid, 1:265.

Based on the MRI findings, histology report and positive serology a diagnosis of hydatid disease of the pelvis was made and she was referred to the Infectious disease unit for further management where she was started on treatment with Albendazole. Further investigations showed no evidence of liver or lung disease.

Despite the treatment with albendazole, symptoms persisted and a MRI on 24/9/99 showed progression of cysts in the right ilium and thigh with hip joint effusion. Although the cysts in the thigh region were thought not be of hydatid origin, due to the pain she was having she underwent an operation and had removal of two cysts one along the lateral side of rectus femoris and the other one deep to gluteus medius on 25/10/99. The operation was covered with Praziquental and Albendazole and she was discharged home on Albendazole. Histology again confirmed the diagnosis of hydatid disease. She was reviewed in the clinic on 6/6/00 and had an MRI on 27/6/00 as she had increasing pain in the right hip and she stopped taking albendazole as she was having hair loss. MRI was compared with the old one and was reported that the cystic lesion in the right ilium was getting bigger.

**Figure 1:**
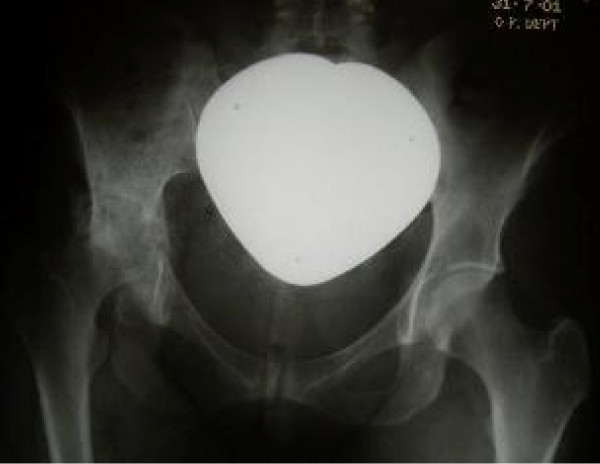
**Radiograph showing involvement of Right ilium and hip by the Hydatid disease**.

On 8/8/00 she returned with severe right hip pain with reduced walking distance of only one hundred yards. She now had limitations of hip flexion and rotations. X-rays showed reduction of right hip joint space with changes in the right ilium (Figure 1). Chest x-ray did not reveal any abnormality. It was thought that hip replacement was too risky and she was advised to continue Albendazole.

**Figure 2:**
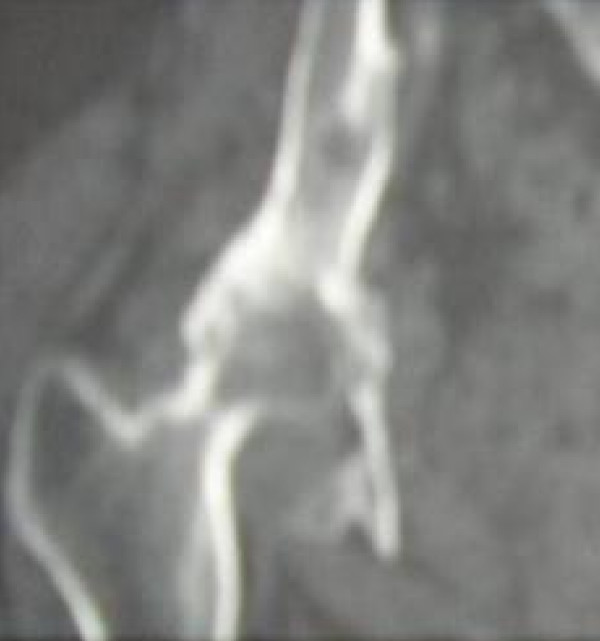
**CT scan showing destruction of the hip with superior migration of the femoral head into the iliac bone affected by the hydatid disease**.

**Figure 3:**
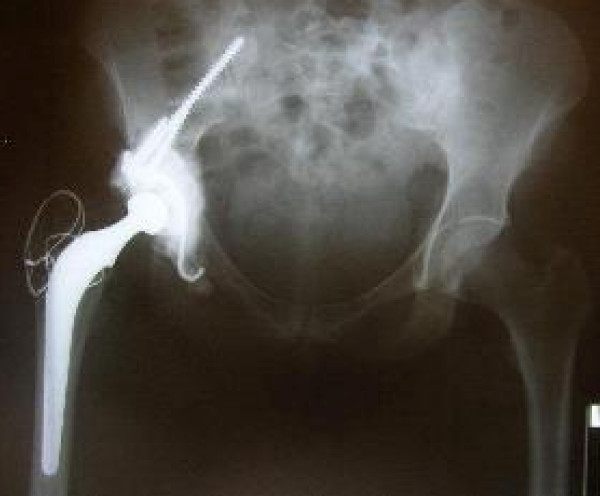
**Radiograph after Right total hip replacement**.

**Figure 4:**
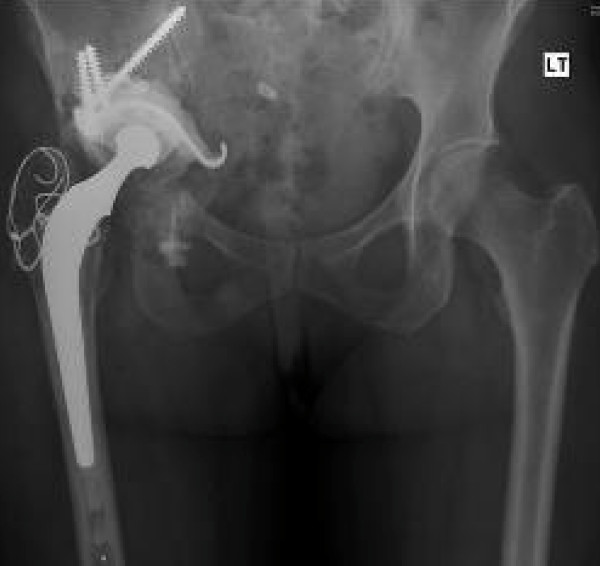
**Radiograph showing loose acetabular component 80 months after index surgery**.

**Figure 5:**
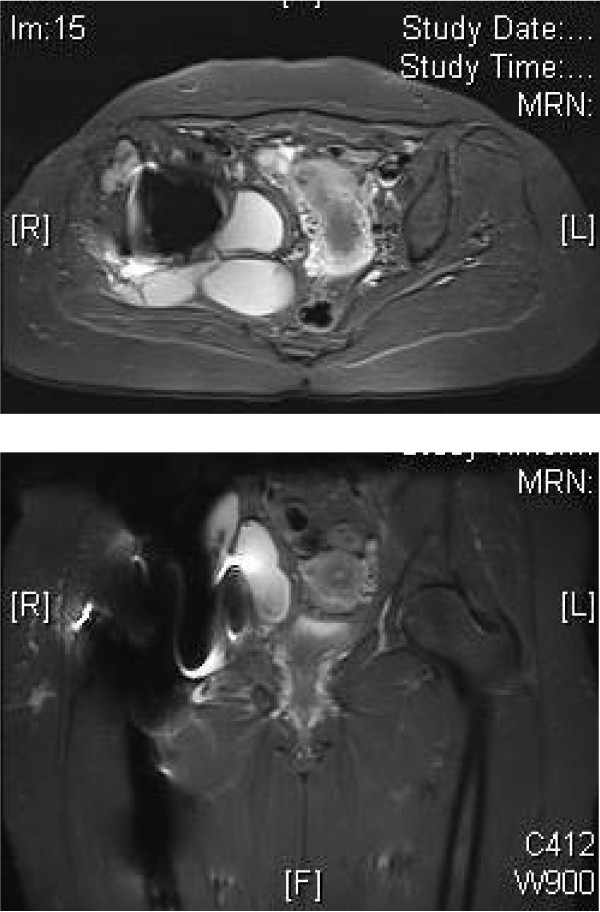
**MRI scans showing recurrent Hydatid cysts**.

On 21/6/01 she was noted to have a fixed flexion deformity of the hip of 40 degrees and further flexion and rotations were painful. X-ray showed lytic lesion of the ilium and 2 centimetre proximal migration of the femoral head into the infected bone (Figure 2). It was felt that her symptoms had reached a stage where surgery was the only option. After considering different surgical options including hindquarter amputation, internal hemipelvectomy and total hip replacement along with the risk of an anaphylactic reaction if she was to have surgery, she underwent a cemented total hip replacement (Figure 3) and subtotal excision of hydatid cysts on 5/2/2002 with appropriate precautions. Postoperative recovery was uneventful. She was pain free and she was able to walk unaided within three months following the total hip replacement. Annual review with radiographs and MRI showed gradual recurrence of the cysts despite the ongoing Albendazole treatment. She returned again on 8/7/08, with increasing pain in the right hip. X-ray has shown that loosening of the acetabular component of the total hip replacement (Figure 4). MRI showed extensive cystic changes all around the hip (Figure 5). She was given the option of having a hemi pelvic resection or an acetabular reconstruction leaving the hydatid cysts. She opted for the acetabular reconstruction option due to the potential functional loss associated with hemi pelvic resection. On 24/10/08 she underwent a customised Ice-cream cone hemi pelvic replacement [Stanmore Implants Worldwide]. During the operation, cysts were seen beneath the deep fascia and an attempt was made to remove all the visible cysts. The acetabular component was loose and it was easily removed. There was a complete loss of posterior column with discontinuity of ilium from pelvis. Curettage and excision of all the visible cysts was performed. A small Ice-cream cone prosthesis was carefully inserted into the remaining ilium and the whole construct was surrounded with bone cement. A tripolar cup was inserted with a cemented 50 mm Serc liner and bipolar head articulated with existing femoral component. She was given Praziquental for 3 days and Albendazole for 28 days based on the advice from the infection and tropical medicine team. Post operative recovery was uneventful. She was reviewed on 13/1/09 and X-rays showed that the ice-cream cone replacement was in good alignment. She is walking unaided and she was able to do household work.

**Figure 6:**
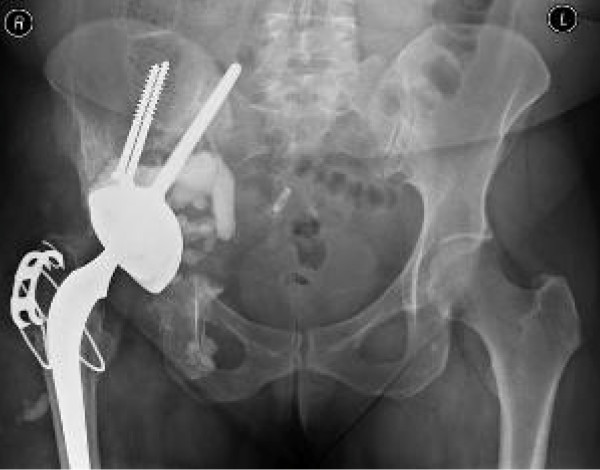
**Radiograph one year after revision of the acetabular component**.

Her recent review (Figure 6) was on 14/10/09 she was pain free and she was able to walk unaided and she could flex her hip to 90 degrees. She has also been advised to take Albendazole for one month every year.

## Discussion

Establishing a diagnosis of bony Hydatid disease can be difficult especially in countries where the disease is extremely rare. Bony hydatid disease is rare even in endemic areas. Symptoms and signs are often mistaken for bacterial infection and the presence of organisms like Propionibacterium and coagulase negative staphylococci from the biopsy material can mask the underlying hydatid disease. In our case, we encountered an extended bone and soft tissue disease with no signs of systemic infection but a history of multiple recurrences. Extensive involvement of the ilium and destruction of the hip joint not responding to Albendazole necessitated surgical intervention in the form of right total hip replacement which lasted for 80 months. Due to the recurrence of the Hydatid cyst the acetabular component became loose and symptomatic and it was revised. To our knowledge, no such case has been reported in the worldwide literature though there are few reports of musculoskeletal Hydatid disease. (Table 1).

**Table 1:** Reported sites of Hydatid disease of the musculoskeletal system.

Author	No*	Site of infection
Bal et al [1]	3	bone
Bellil et al [2]	6	bone
Merkle et al [7]	8	Iliopsoas, left adductor musculature, left femur, left gluteus medius muscle, musculature of right upper leg
Metcalf JE [8]	1	bone - humerus
Natarajan MV [9]	3	bone - femur
Torricelli et al [11]	14	bone infection with adjacent soft tissue involvement in 12 cases
Dahniya et al [3]	7	5 bone infections without soft tissue involvement, 2 primary intramuscular (lt. shoulder, rectus femoris and vastus lateralis)

* Number of patients

Determining the ideal therapeutic approach for a musculoskeletal hydatid cyst not responding to medical treatment can be quite challenging. Conservative treatment, complete excision and simple drainage have all been suggested as treatment options [11]. Hydatid disease progresses slowly and is rarely life-threatening, especially when located in the soft tissue or muscles, thus supporting a conservative therapeutic approach. However, if the Hydatid disease causes profound disabilities or mobilization problems, complete cystopericystectomy and even total joint replacement becomes an option. Radical surgical excision is especially indicated in cases of unilocular manifestations as only this method offers hope of permanent cure [10]. Therapeutic dilemmas could arise in cases of extended disease with many muscles or muscle layers in different sites of the body which are communicating *via *fistulas. Communication between lesions should always be suspected and revealed, even if primary and daughter cysts are distant. Complete surgical treatment should include the primary lesion, the daughter cysts and the communicating fistulas as a whole specimen. Bony pelvis is a difficult location for radical surgical excision of the Hydatid cyst and the morbidity of a Hindquarter amputation can be considerable. Subtotal excision of the cyst and joint replacement is an acceptable option based on our case report. Subtotal excision of the Hydatid cyst of the pelvis and a hip replacement can be durable providing adequate function. Patients should be carefully monitored for cyst recurrence and component loosening. Loose components due to recurrent cysts can be successfully revised to provide good clinical outcome.

## Competing interests

The authors declare that they have no competing interests.

## Authors' contributions

All authors contributed to the article. All authors have read and approved the final manuscript.
